# Gene therapy in advanced metachromatic leukodystrophy: tempering expectations

**DOI:** 10.1093/procel/pwae065

**Published:** 2024-11-28

**Authors:** Daphne H Schoenmakers, Shanice Beerepoot, Laura A Adang, Marije A B C Asbreuk, Caroline G Bergner, Annette E Bley, Jaap-Jan Boelens, Valeria Calbi, Alejandra Darling, Erik Eklund, Ángeles García Cazorla, Sabine W Grønborg, Samuel Groeschel, Peter M van Hasselt, Carla E M Hollak, Claire Horgan, Simon Jones, Tom de Koning, Lucia Laugwitz, Caroline Lindemans, Pascal Martin, Fanny Mochel, Andreas Øberg, Dipak Ram, Caroline Sevin, Ludger Schöls, Ayelet Zerem, Nicole I Wolf, Francesca Fumagalli

**Affiliations:** Department of Child Neurology, Amsterdam Leukodystrophy Center, Amsterdam UMC Location Vrije Universiteit Amsterdam, Emma’s Children’s Hospital, Boelelaan 1117, Amsterdam, The Netherlands; Amsterdam UMC, Vrije Universiteit Amsterdam, Department of Cellular and Molecular Mechanisms, Amsterdam Neuroscience, De Boelelaan 1117, Amsterdam, The Netherlands; Medicine for Society, Platform at Amsterdam UMC Location University of Amsterdam, Meibergdreef 9, Amsterdam, The Netherlands; Department of Child Neurology, Amsterdam Leukodystrophy Center, Amsterdam UMC Location Vrije Universiteit Amsterdam, Emma’s Children’s Hospital, Boelelaan 1117, Amsterdam, The Netherlands; Amsterdam UMC, Vrije Universiteit Amsterdam, Department of Cellular and Molecular Mechanisms, Amsterdam Neuroscience, De Boelelaan 1117, Amsterdam, The Netherlands; Division of Neurology, Children’s Hospital of Philadelphia, Philadelphia, PA 19104, United States; Department of Child Neurology, Amsterdam Leukodystrophy Center, Amsterdam UMC Location Vrije Universiteit Amsterdam, Emma’s Children’s Hospital, Boelelaan 1117, Amsterdam, The Netherlands; Amsterdam UMC, Vrije Universiteit Amsterdam, Department of Cellular and Molecular Mechanisms, Amsterdam Neuroscience, De Boelelaan 1117, Amsterdam, The Netherlands; Medicine for Society, Platform at Amsterdam UMC Location University of Amsterdam, Meibergdreef 9, Amsterdam, The Netherlands; Leukodystrophy Center, Clinic for Neurology, University hospital Leipzig, 04103 Leipzig, Germany; University Children’s Hospital, University Medical Center Hamburg Eppendorf, 20251 Hamburg, Germany; Department of Pediatrics, Stem Cell Transplantation and Cellular Therapies Program, Memorial Sloan Kettering Cancer Center, New York, NY 10065, United States; San Raffaele Telethon Institute for Gene Therapy (SR-TIGET), Pediatric Immunohematology Unit, IRCCS San Raffaele Scientific Institute, Via Olgettina, 60, Milan 20132, Italy; Metabolic Unit, Neurology Department, Sant Joan de Déu Children´s Hospital, Barcelona, Spain; Section for Pediatric Neurology, Skåne University Hospital and Clinical Sciences, Lund University, Lund 221 84, Sweden; Metabolic Unit, Neurology Department, Sant Joan de Déu Children´s Hospital, Barcelona, Spain; Department of Pediatrics and Adolescent Medicine and Department of Clinical Genetics, Center for Inherited Metabolic Diseases, Copenhagen University Hospital Rigshospitalet, Copenhagen, Denmark; Department of Paediatric Neurology and Developmental Medicine, University Children’s Hospital, Tübingen, Germany; Department of Metabolic Diseases, University Medical Center Utrecht, Utrecht, The Netherlands; Medicine for Society, Platform at Amsterdam UMC Location University of Amsterdam, Meibergdreef 9, Amsterdam, The Netherlands; Department of Endocrinology and Metabolism, Amsterdam UMC Location AMC, Amsterdam UMC, Meibergdreef 9, University of Amsterdam, Amsterdam, The Netherlands; Department of Paediatric Bone Marrow Transplant and Cellular Therapy, Royal Manchester Children’s Hospital, Manchester University NHS Foundation Trust, United Kingdom; Genomic Medicine, St Mary’s Hospital, Manchester University NHS Foundation Trust, United Kingdom; Section for Pediatric Neurology, Skåne University Hospital and Clinical Sciences, Lund University, Lund 221 84, Sweden; Neuropediatrics, General Pediatrics, Diabetology, Endocrinology and Social Pediatrics, University of Tuebingen, University Hospital Tübingen, Tübingen 72016, Germany; Institute for Medical Genetics and Applied Genomics, University of Tübingen, Tübingen 72070, Germany; Department of Pediatric Hematopoietic Stem Cell Transplantation, UMC Utrecht and Princess Maxima Center, The Netherlands; Department of Neurology and Epileptology, Hertie Institute for Clinical Brain Research, University of Tübingen, Tübingen 72070, Germany; Sorbonne Université, Institut du Cerveau, Inserm, CNRS, AP-HP, Paris, France; Department of Genetics, AP-HP, Hôpital Pitié-Salpêtrière, DMU BioGeM, Paris, France; Norwegian National Unit for Newborn Screening, Division of Pediatric and Adolescent Medicine, Oslo University Hospital, Norway; Department of Paediatric Neurology, Royal Manchester Children’s Hospital, United Kingdom; Pediatric Neurology Department, Reference Center for Leukodystrophies, Hôpital Bicêtre, Le Kremlin Bicêtre, France; Department of Neurology and Hertie Institute for Clinical Brain Research, University of Tübingen, Tübingen 72070, Germany; German Center of Neurodegenerative Diseases (DZNE), Tübingen, Germany; Faculty of Medicine and Health Sciences, Tel Aviv Sourasky Medical Center, Pediatric Neurology Institute, Dana-Dwek Children’s Hospital, Tel Aviv University, Tel Aviv, Israel; Department of Child Neurology, Amsterdam Leukodystrophy Center, Amsterdam UMC Location Vrije Universiteit Amsterdam, Emma’s Children’s Hospital, Boelelaan 1117, Amsterdam, The Netherlands; Amsterdam UMC, Vrije Universiteit Amsterdam, Department of Cellular and Molecular Mechanisms, Amsterdam Neuroscience, De Boelelaan 1117, Amsterdam, The Netherlands; Pediatric Immunohematology Unit and Neurology and Neurophysiology Unit, San Raffaele Telethon Institute for Gene Therapy (SR-TIGET), IRCCS San Raffaele Scientific Institute, Via Olgettina, 60, Milan 20132, Italy

**Figure 1.**
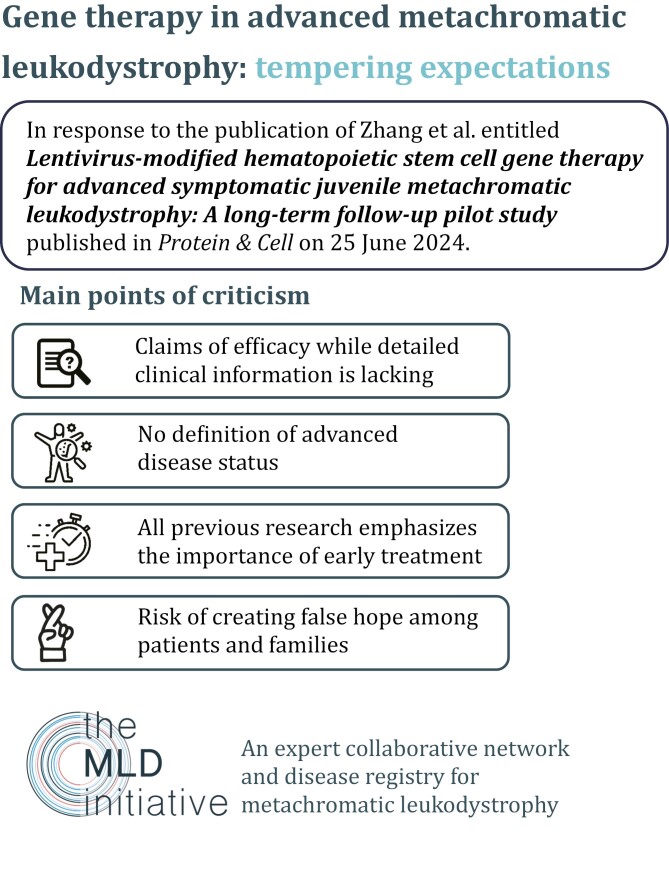
Critical response to the publication of Zhang et al. regarding gene therapy in advanced MLD.

Recently Zhang et al. (2024) published their study entitled “*Lentivirus-modified hematopoietic stem cell gene therapy for advanced symptomatic juvenile metachromatic leukodystrophy: A long-term follow-up pilot study.*” The authors present three metachromatic leukodystrophy (MLD) patients treated with gene therapy and claim stabilization or even improvement, despite advanced symptomatic disease stage. The metachromatic leukodystrophy initiative (MLDi) (Schoenmakers et al., 2022), an international collaborative network and registry for MLD, urges caution in interpreting these results, as the evidence raises several critical concerns. These claims risk fostering false hope among MLD patients and their families, particularly given the significant gaps in the data provided (Fig. 1).

The authors suggest beneficial outcomes of gene therapy in advanced MLD. Two of the three patients (MLD01 and MLD02) presented were already clearly affected at the time of treatment, exhibiting symptoms indicating advanced disease, such as dysphagia, urinary incontinence, and loss of walking. Based on an increased functional independence measure (FIM) score and/or gross motor function classification for MLD (GMFC-MLD) the authors suggest considerable improvement, e.g., walking with quality and performance normal for age. However, in addition to this composite and crude clinical score, detailed clinical information about, e.g., cognition, gross- and fine motor function, eating and drinking ability, and speech is necessary to comprehensively assess the clinical status of the patients and substantiate the claim of neurological improvement. The authors interpret improved arylsulfatase A (ARSA) activity as a treatment benefit. This biochemical characteristic implies technical treatment success, but should not be confused with clinical benefit.

The third treated patient (MLD03) was diagnosed pre-symptomatically at age 1.6 years following family screening and cannot be considered an advanced symptomatic MLD patient. The near-normal Magnetic Resonance Imaging (MRI) at diagnosis and maximum clinical scores advocate for an early disease stage at baseline. The described muscle weakness may be explained by peripheral neuropathy, but no information on electro-neurophysiological tests is given. It is common that peripheral neuropathy appears early in the disease course of MLD and may even be present years before the central manifestation of the disease (Beerepoot et al., 2019). Treatment before developing central nervous system symptoms is generally followed by good clinical outcomes (Boucher et al., 2015; Fumagalli et al., 2022; Groeschel et al., 2016; van Rappard et al., 2016).

The article lacks crucial details, such as detailed inclusion criteria defining “advanced disease status,” the total number of treated patients, and outcomes of other treated patients. This information is essential to understand the efficacy and safety of a new treatment. Moreover, the reported *in vivo* vector copy numbers appear suboptimal for achieving enzyme activity overexpression necessary for significant clinical benefit.

Previous research emphasizes that severe nervous system damage is irreversible, and full recovery of lost neurological function is unlikely (Fumagalli et al., 2021). The impressive improvement from GMFC-MLD level 4 to level 0 in MLD01 is questionable, particularly considering the extensive damage on baseline MRI. Regaining normal walking in quality and performance (GMFC-MLD 0) after complete loss of upright mobility (GMFC-MLD 4) is very unlikely if caused by neurological damage (cerebellar, spasticity, or neuropathy). This has never been observed in previous *ex vivo* gene therapy trials for MLD (Fumagalli et al., 2022), highlighting the need for caution in interpreting these results.

Several studies reporting outcomes of allogeneic hematopoietic stem cell transplantation have shown the importance of treating before severe symptoms occur (Boucher et al., 2015; Groeschel et al., 2016; van Rappard et al., 2016). The conditioning regimen with chemotherapy may even trigger deterioration in advanced disease stages (Beschle et al., 2020). The past years of experience with the use of atidarsagene autotemcel (Libmeldy^TM^), the authorized lentiviral gene therapy for MLD in the European Union and the USA, have confirmed this. When patients are too advanced, gene therapy is not beneficial (Fumagalli et al., 2022). During the trial of Fumagalli et al. (2022), the eligibility criteria were even amended to avoid inclusion of severely affected juvenile patients. Nowadays, the eligibility criteria adopted by experts include the ability to walk without support (GMFC-MLD < 2) and substantial residual cognitive function (total intelligence quotient ≥ 85) (Schoenmakers et al., 2024). We acknowledge that treatment decisions for borderline patients are difficult. Especially late-juvenile and adult MLD patients can present with an insidious onset and slow decline. Careful consideration of potential risks associated with treatment, along with the fact that the beneficial effects of autologous and allogeneic stem cell therapy can be expected after 6–12 months, is essential in treatment decisions.

To conclude, the message portrayed in the study of Zhang et al. is not in line with current best practices for the management of MLD patients (Fumagalli et al., 2022; Laugwitz et al., 2024b) and provides insufficient detail to judge efficacy and safety of this new and invasive treatment. We acknowledge the significant unmet need for treatments for late-juvenile and adult MLD, as well as for advanced disease stages. Fortunately, atidarsagene autotemcel is currently being investigated in early-symptomatic late-juvenile patients (NCT04283227). Future treatments in advanced disease stages will at best be able to modify the disease course, but not to achieve a cure or significant improvement. To identify patients in time to guarantee successful treatment, newborn screening is the best option (Laugwitz et al., 2024b, 2024a).
